# Supplementary material for: A Comparison between Core Stability Exercises and Muscle Thickness Using Two Different Activation Maneuvers
Source: J Funct Morphol Kinesiol. 2024 Apr 11;9(2):70. doi: 10.3390/jfmk9020070 (PMC11036226; doi:10.3390/jfmk9020070)
Supplement: Supplementary file 1 [file jfmk-09-00070-s001.zip › jfmk-2936622-supplementary.pdf]

Exercise protocol

A) *Elbow Plank and reach.* Begin in an elbow plank position with your feet slightly wider than your hips to create more stability. Your elbows should be directly under your shoulders and your forearms facing forward. Ensure that your head is relaxed and looking at the floor. Upon receiving instructions from the examiner, inhale and activate your core muscles using either the hollowing or bracing method while exhaling. Keep your torso straight and rigid, ensuring your body forms a straight line from your ears to your toes without sagging or bending. When performing the arm extension, carefully shift your weight to your right forearm or palm and extend your left arm straight out in front of you. Maintain this position for 10 seconds or until instructed by the researcher to return to the starting position.

B) *Bird Dog Exercise.* Start in the tabletop position on all fours with your knees under your hips and your hands under your shoulders. Draw your shoulder blades together and maintain a neutral spine by engaging your abdominal and back muscles. Upon receiving instructions from the examiner, inhale and activate your core muscles using either the hollowing or bracing method while exhaling. Maintain a position with your left arm raised and your right leg lifted, ensuring that your shoulders and hips remain parallel to the floor. Keep the back of your neck elongated and your chin tucked in towards your chest, looking downwards at the floor. Hold this position for 10 seconds or until instructed by the researcher to return to the starting position.

C) *Beast Crawl Exercise*. Begin on your hands and knees with your toes tucked under and digging down into the ground. The hands should be shoulder width apart, while the knees and feet are hip width apart. Bring your left hand and left foot ten centimeters in front of your right hand and right foot. Keep your torso straight and rigid. Upon receiving instructions from the examiner, inhale and activate your core muscles using either the hollowing or bracing method while exhaling. The beast hold starts when you engage your abs, squeeze your glutes, and raise your knees exactly four to five centimeters off the ground. Hold this position for 10 seconds or until instructed by the researcher to return to the starting position.

D) *Pilates Toe Tap Exercise.* To perform this exercise, lie on your back on a flat surface and bring your knees up to a 90-degree angle, also known as the tabletop position. It is important to engage your abdominal muscles to maintain stability throughout the exercise. Upon receiving instructions from the examiner, inhale and activate your core muscles using either the hollowing or bracing method while exhaling. Lower your left foot slowly to the ground while maintaining a 90-degree angle in your knee. Lightly touch the floor with your toes and hold this position for 10 seconds or until instructed by the researcher to return to the starting position.

E) *Dead Bug Exercise.* Start by lying on your back on a flat surface. Raise both arms in a vertical position with your body and also raise your knees in the air at a 90-degree angle, also known as the tabletop position. It is important to use your core muscles to maintain stability throughout the exercise. As instructed by the assessor, inhale and engage your core muscles using either the hollowing or bracing method as you exhale. Lower your right arm and left leg parallel to the floor, keeping your shoulders and hips parallel to floor. Stretch the back of your neck and place your chin on your chest, facing the floor, and hold this position for 10 seconds or until instructed to return to the starting position by the assessor.

F) *Shoulder Bridge Exercise with One Leg Extended*. Start by lying on your back with your legs bent and hip width apart. Your arms should be by your side, palms down. As instructed by the assessor, inhale and engage your core muscles using either the hollowing or squeezing method as you exhale to lift your pelvis and hips, creating a diagonal line from knee to shoulder. Stretch one leg straight out. Hold this position for 10 seconds or until instructed to return to the starting position by the assessor.

G) *Side Plank with Extended Arm*. Start on your right side with your body fully extended. Lift your body off the ground and balance your weight between your forearm and the side of your foot. Your right arm should be directly under your shoulder with the hand on the floor and the arm extended. Keep your torso straight and rigid and your body in a straight line from your ears to your toes, without sagging or bending. As instructed by the assessor, inhale and as you exhale, engage your core muscles using either the hollowing or squeezing method. Gently shift your weight to your right forearm (or palm). Stretch your left arm straight out, vertically to your body. Hold this position for 10 seconds and return to the starting position.

All of the above exercises can be modified to increase or decrease the difficulty, depending on the individual's strength and fitness level. As with any exercise, it is important to maintain proper form to avoid injury and maximize the benefits of the exercise.
